# Peripapillary hyperreflective ovoid mass‐like structures: Multimodal imaging—A review

**DOI:** 10.1111/ceo.14182

**Published:** 2022-11-06

**Authors:** Rachael C. Heath Jeffery, Fred K. Chen

**Affiliations:** ^1^ Centre for Ophthalmology and Visual Science (incorporating Lions Eye Institute) The University of Western Australia Nedlands Western Australia Australia; ^2^ Royal Victorian Eye and Ear Hospital (Centre for Eye Research Australia) East Melbourne Victoria Australia; ^3^ Ophthalmology, Department of Surgery The University of Melbourne East Melbourne Victoria Australia; ^4^ Department of Ophthalmology Royal Perth Hospital Western Australia Australia

**Keywords:** optic disc drusen, optic disc oedema, optic nerve, optical coherence tomography, peripapillary hyperreflective ovoid mass‐like structures

## Abstract

Peripapillary hyperreflective ovoid mass‐like structures (PHOMS) are a laterally bulging herniation of distended axons into the peripapillary region above the level of Bruch's membrane opening. Increased use of enhanced depth imaging‐optical coherence tomography (EDI‐OCT) in our evaluation of the optic nerve head (ONH) and greater recognition of the vast range of optic nerve pathologies with which PHOMS is associated provides convincing evidence that PHOMS is not just buried optic disc drusen (ODD) as previously described. The frequent coexistence of PHOMS with ODD, papilloedema, anterior ischaemic optic neuropathy, tilted optic disc syndrome, inflammatory demyelinating disorders and other diseases associated with axoplasmic stasis provides insight into its underlying pathophysiology. The present review will discuss the role of key imaging modalities in the differential diagnosis of PHOMS, explore the current literature on the relationship between PHOMS and common neuro‐ophthalmic conditions, and highlight the gaps in our knowledge, with respect to disease classification and prognosis, to pave the way for future directions of research.

## 1 HISTORICAL BACKGROUND

The *Optic Disc Drusen Studies* (ODDS) *Consortium*, established in 2015 to provide an international forum for optic disc drusen (ODD) research into ODD‐related vision loss, defined peripapillary hyperreflective ovoid mass‐like structures (PHOMS) as a sign of axoplasmic stasis on optical coherence tomography (OCT).^1^ Importantly, PHOMS is an OCT diagnosis and it is not a precursor or subtype of ODD.^1^ It was hypothesized that PHOMS represented a lateral herniation of distended retinal ganglion cell axons into the peripapillary region, wedged between the peripapillary nerve fibre layer and Bruch's membrane, forming a torus or doughnut‐like structure around the disc margin.^2^

Prior to 2018, peripapillary subretinal lesions identical to PHOMS were labelled ‘buried ODD’ in eyes with pseudopapilloedema.^3^ These ‘buried ODD’ and ‘nerve fiber herniations’ were also reported to co‐exist with papilloedema,^4^ tilted disc syndrome^5^ and optic nerve tumours^6^ prior to the ODDS Consortium Statement. Recent observations of an association between PHOMS and central nervous system (CNS) demyelination,^7^, ^8^ ischaemic optic neuropathy and retinal vein occlusions (RVO)^9^ provides further evidence that this imaging feature is a common endpoint of axoplasmic stasis as opposed to a non‐calcified subtype of ODD. Despite many of the above entities lacking clinicopathological correlation, histopathology of eyes with papilledema have demonstrated herniated retinal nerve fibre layers (RNFL) that correlate with the location of PHOMS and resemble similar features to that seen on enhanced depth imaging(EDI)‐OCT.^10^ These lateral RNFL protrusions have also been reported in histopathological specimens of ODD (described as retinal scarring),^11^, ^12^ nonarteritic anterior ischaemic optic neuropathy (NA‐AION),^13^ central retinal vein occlusion (CRVO),^14^ tilted optic disc syndrome^15^ and acute demyelinating optic neuritis in a guinea pig model.^16^ Given PHOMS acts as a biomarker of axoplasmic stasis and may harbour a potentially life and sight‐threatening neurologic and ophthalmic disease, it is important to recognise this imaging sign and to avoid confusion with ODD.

Herein, we review the multimodal imaging features of PHOMS and discuss this sign across a broad spectrum of disease presentations. Furthermore, we highlight current gaps in our knowledge regarding the relevance of PHOMS with respect to disease classification, prognosis and as a potential clinical trials endpoint measure.

## 2 MULTIMODAL IMAGING FEATURES OF PHOMS

The earliest OCT report of a PHOMS‐like subretinal hyperreflective lesion was described in 45 patients with presumed ‘buried ODD’.^4^ In one of 15 patients with optic disc oedema, due to various causes, a co‐existing PHOMS‐like lesion was mistakenly labelled as an incidental ‘buried ODD’. In a subsequent case series, these PHOMS‐like lesions were classified as one of three types of ODD, although the authors acknowledged their uncertainty of whether the histological correlate of this subretinal lesion was indeed composed of drusen material given the lack of calcification.^17^ In a letter to the authors, Malmqvist et al. suggested that such ‘peripapillary drusen’ should not be considered within the spectrum of ODD since they do not have the typical hyporeflective core and hyper‐reflective border, nor do they demonstrate hyperautofluorescence or hyper‐echogenicity on ultrasound (US).^18^ The ODDS Consortium used OCT images from 38 patients with ODD to develop a consensus approach to identify known ODD and classify lesions that were not consistent with ODD. The term PHOMS was initially used for these subretinal hyperreflective mass lesions with specific multimodal imaging features as discussed below and the definition was further refined in a subsequent multi‐rater validation study.^19^

### 2.1 Optical coherence tomography features

**FIGURE 1:**
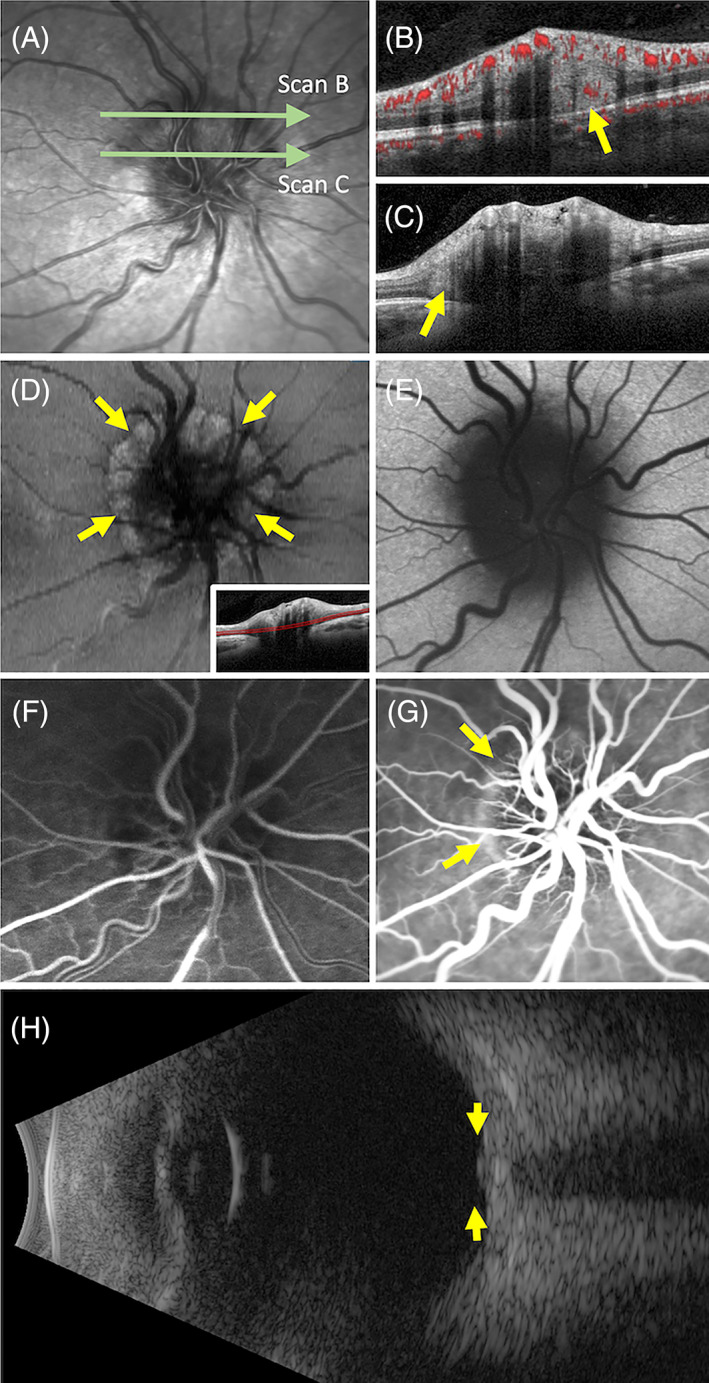
A peripapillary hyperreflective ovoid mass‐like structure (PHOMS) in a 29‐year‐old female presenting with bilateral asymmetric optic nerve head (ONH) elevation (A). OCTA showed flow signal within the hyperreflective lesion marked by yellow arrow (B). The internal hyperreflectivity is homogenous (C). *En face* reconstruction of intensity projection stable just above the level of the Bruch's membrane (inset) showed a hyperreflective structure (PHOMS) encircling the disc (D) as marked by yellow arrow. This is hypoautofluorescent (E). Early (F) and late (G) phase fluorescein angiography showed staining of the PHOMS. Ultrasound showed an elevated ONH but no hyperechogenicity (H).

PHOMS are defined by their location, internal optical features, shape and effect on surrounding tissues.^2^, ^19^ A typical PHOMS, as shown on OCT, is illustrated in Figure 1.

1. Location: peripapillary as a complete or partial torus structure that correlates clinically with the extent of the obscured disc margin. PHOMS are wedged between Bruch's membrane and the RNFL, abutting the peripapillary outer nuclear layer. An intensity projection *en face* image, reconstructed from OCT volume scans, can be used to determine the circumferential extent of PHOMS around the disc margin (Figure 1D).
2. Internal optical features: diffusely hyperreflective internally with a similar reflectivity to the RNFL (Figure 1C). Internal vascular flow signals have been observed by two independent groups using OCT‐angiography (OCTA) suggesting an intrinsic vascular supply (Figure 1B).^20^, ^21^ However, Kim et al. reported a lack of flow signal as a supporting argument for PHOMS being composed of extracellular drusen material.^22^ Mezad‐Koursh et al. also found 94% of PHOMS showing internal hyperreflective foci which they proposed were early calcium deposits despite the lack of supporting histological evidence.
3. Shape: on cross‐sectional view, PHOMS appears ovoid without any associated complex geometric shapes. The location of the OCT slice determines the morphology of the ovoid structure. In a cross‐sectional study of PHOMS, the mean height and width of this ovoid lesion was 399 and 721 μm respectively.^21^
4. Effect on surrounding tissues: The plane of Bruch's membrane is unaffected in contrast to the inner retinal layers (2 or more) which are displaced upwards to give the impression of a blurred and elevated disc margin. PHOMS does not invade or infiltrate peripapillary retinal tissue but rather acts like a space occupying lesion producing a ‘ski slope’‐like deflection of the inner retinal surface, slanting down and away from the disc margin.

**FIGURE 2:**
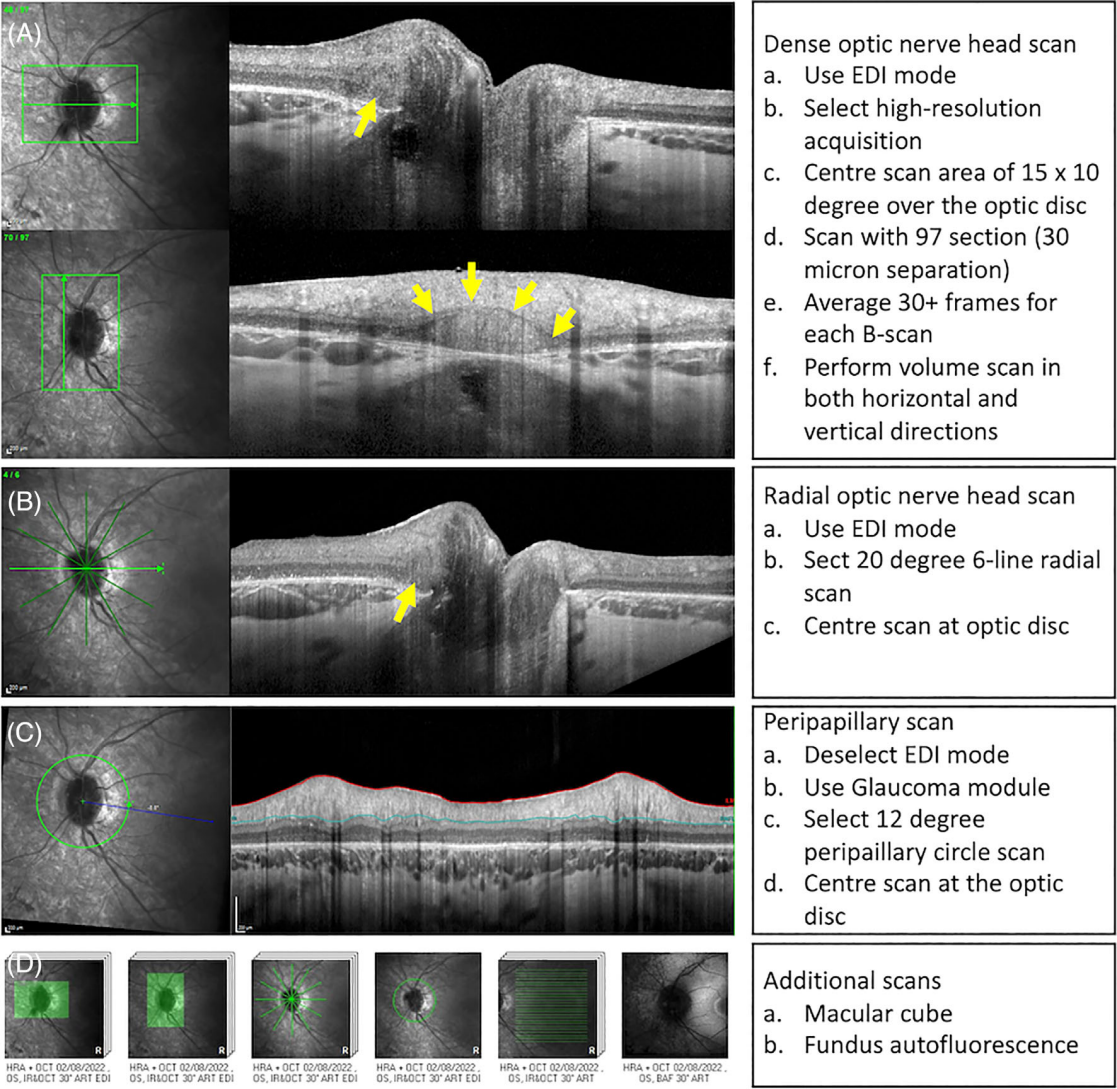
Optic disc drusen (ODD) studies consortium recommended a disc imaging protocol for the detection of disc drusen and associated structures. Dense optic nerve head (ONH) scan (A) should be acquired in both horizontal and vertical orientations in the enhanced depth imaging (EDI) mode. High resolution 6 radial scan (B) is also recommended in addition to the standard peripapillary nerve fibre circle scan in the glaucoma mode (C). Additional macular OCT and disc centred fundus autofluorescence imaging is recommended (D). This 21‐year‐old female has PHOMS (yellow arrow) in association with ODD in Usher syndrome type 2.

The ODDS Consortium recommended the use of a dedicated optic disc OCT scanning protocol in addition to the commonly used peripapillary RNFL circle scan in the assessment of ODD and visualisation of PHOMS. The OCT and EDI‐OCT protocols for disc imaging are shown in Figure 2. Notably these are the minimum requirements for image acquisition on any commercial OCT device that utilises spectral domain technology and offers an enhanced depth imaging mode. Additional single line scans manually positioned at regions of interest and combining OCT with fundus autofluorescence (FAF) imaging should also be considered. A standard macular scan of 20° × 20° does not incorporate the optic nerve head (ONH). In clinical practice, a nasally decentred macular raster scan of 30° × 25° captures the entire ONH and fovea, thus allowing PHOMS to be identified prior to committing to the comprehensive optic disc scanning protocol where more detailed imaging is required to characterise PHOMS. Most commercial OCT devices offer an OCTA module. Future protocol updates from the ODDS should include OCTA as the imaging and analytical approach to OCTA data matures and becomes more standardised.^23^

OCTA provides visualisation of the internal vascularity of PHOMS, peripapillary vascular network and quantification of the retinal microvasculature. Ahn et al. found vessel densities measured in the papillary and peripapillary region were significantly lower in children harbouring large PHOMS (height ≥ 500 μm) as compared to controls.^24^ However, none of the vascular parameters in the small (height < 300 μm) and medium (300–500 μm) sized PHOMS eyes were statistically different from controls. Large PHOMS were more frequently associated with ODD than small PHOMS. Given the reduced flow index and ONH vascular density reported in ODD patients as compared to normal controls,^25^ it remains to be seen if these microvascular alterations seen in larger PHOMS is secondary to the coexisting ODD or related to the effect of PHOMS abutting on the adjacent peripapillary RNFL. Further work on quantifying the intrinsic vascular structures in PHOMS is needed.

### 2.2 Other imaging modalities

Near‐infrared reflectance (NIR) imaging has demonstrated a hyporeflective ring nasal to the disc margin, correlating with the exact location of the PHOMS edge.^26^ However, it is not clear how often this feature is also seen in other causes of disc swelling without PHOMS. In contrast to ODD, PHOMS typically do not show a hyper‐AF signal on short wavelength FAF imaging.^21^ Studies that described ‘peripapillary ODD’ also noted that these PHOMS‐like lesions lack the hyper‐AF typically seen in traditional ODD.^17^ Yan et al. reported a hypo‐AF signal in a ring or crescent around the disc margin, corresponding to the extent of PHOMS, attributed to an absence of the underlying retinal pigment epithelium (Figure 1E).^27^ In contrast Mezad‐Koursh et al. found small hyper‐AF spots in 47% of the eyes with PHOMS with good inter‐observer reliability.^26^ These lesions did not correlate with age or US features. They postulated that these spots might correspond to the small hyperreflective foci within the PHOMS as seen on OCT (see above). To date no fluorescein angiography studies of PHOMS have been performed to determine the permeability of the intrinsic vasculature as observed on OCTA. We report late hyperfluorescence without expansion indicating fluorescein staining of the herniated RNFL tissue (Figure 1F,G).

Alexander Fraser et al. reported PHOMS could only be detected on OCT and not with US or computed tomography (CT).^2^ However, Mezad‐Koursh et al. described distinct echographic features of PHOMS in 78% of their cases where there is a small hyperechogenic structure with no posterior shadowing.^26^ They used a 10 MHz B‐scan and evaluated the size of these hyperechogenic structures using the calliper tool at a gain of 90 dB. The size of the hyperechogenic component displayed parallel to the retina was statistically significantly smaller, located closer to the retinal surface and less echogenic in PHOMS as compared to ODD (Figure 1H). However, the minimal gain to detect the PHOMS was similar to ODD (56 vs. 50 dB). Larger PHOMS on OCT required lower gains to visualise the structures on US and the greater the size of the hyperechogenic structure. When investigating for causes of a swollen ONH, it is important to measure the posterior coat thickness and look for fluid in sub‐Tenon's space and around the optic nerve sheath to exclude posterior scleritis^28^ and increased intracranial pressure,^29^ respectively.

## 3 DISEASES ASSOCIATED WITH PHOMS

PHOMS has been shown to accompany a wide spectrum of ocular and neurologic diseases that share an underlying pathophysiological process of axonal compression and stasis. However, the relative contribution of each of these causes depends on the patient's age. In a case series of 45 eyes from 45 children with PHOMS under the age of 18 years, ODD was seen in 16 eyes (36%).^24^ Although the authors did not mention the underlying causes of PHOMS seen the remaining 29 eyes, they reported a significantly smaller scleral canal diameter and a trend towards a longer axial length suggesting a myopic disc tilt as the primary cause. Another case series of 66 children with PHOMS, Lyu et al. reported these eyes were more myopic (−3.13D vs. −0.95D) and had greater ONH torsion (9.84° vs. 3.71°) compared to a cohort of 36 controls without PHOMS.^30^ Although not labelled with a diagnosis, the children with PHOMS in Lyu et al.'s series may have had varying degrees of myopic disc tilt. A cross sectional study of 37 eyes of 26 adults and children with PHOMS, found the predominant causes were AION (*n* = 12 eyes), optic neuritis (*n* = 11 eyes) and ODD (*n* = 9 eyes).^21^ Other less common associations were tilted disc syndrome, white dot syndrome and macular neovascularisation. As PHOMS was initially described in the setting of ODD, it will be discussed first followed by the association with papilloedema, AION, tilted disc syndrome, inflammatory demyelinating disorders and other miscellaneous retinal and optic nerve diseases.

### 3.1 Optic disc drusen

**FIGURE 3:**
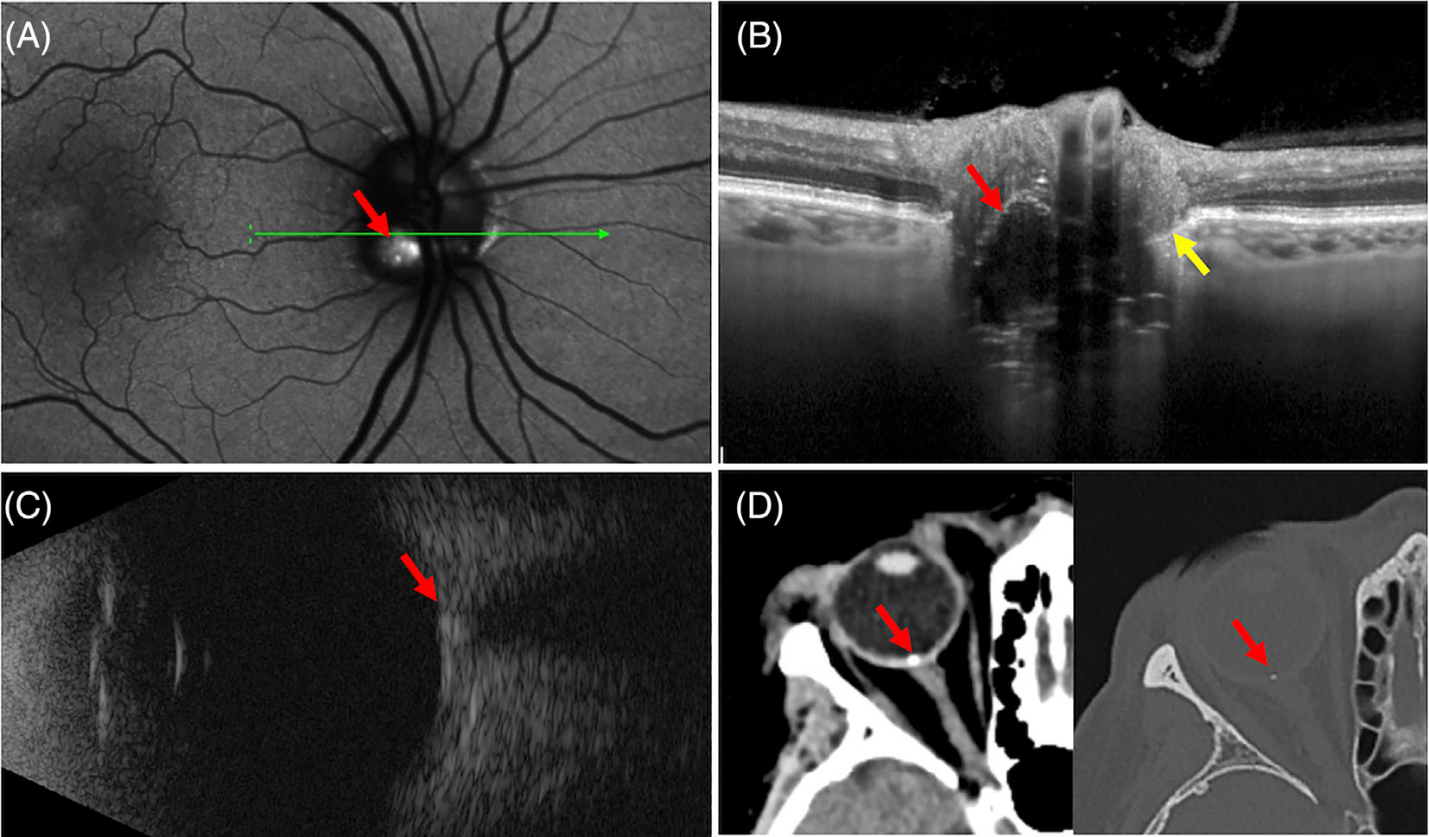
Optic disc drusen (ODD) in a 78‐year‐old female with macular telangiectasia. Fundus autofluorescence showed the typical hyperautofluorescence of the ODD at the disc and foveal hyperautofluorescence due to loss of xanthophyll pigment (A). Corresponding OCT through the ODD showed a deep hyporeflective structure with a hyperreflective superficial border (red arrow) and accompanying PHOMS (yellow arrow, B). The optic nerve head was hyperechogenic on B‐scan ultrasound (C) and bright on computed tomography in the bone window sections indicating calcium (D).

ODD are discrete lesions composed of acellular, extracellular concretions that are usually calcified and located in the unmyelinated RNFL anterior to the lamina cribrosa.^12^, ^31^, ^32^ The prevalence of ODD range from 0.4% in clinical assessment of the paediatric population^33^ to 2.4% in an adult autopsy series.^34^ In a Danish cohort study of 1304 children the prevalence of ODD was 1% using OCT assessment of the ONH.^35^ OCT is an essential imaging modality for detecting ODD in children as they are more likely to be deeper and thus more difficult to detect with fundoscopy or autofluorescence alone. As ODD emerge to the surface with increasing age, they may lead to visual field defects, ischaemic optic neuropathy, disc margin haemorrhage with or without peripapillary choroidal neovascularisation and optic nerve pallor.^36^ The migration of ODD from a buried to a visible location may reduce their effect on axonal compression and RNFL herniation. Histological studies have shown a lateral bulge of the nerve fibres in eyes of ODD, corresponding to PHOMS as seen on the OCT.^12^ Figure 3 shows PHOMS in the setting of a typical ODD characterised by hyper‐AF, hyperechogenicity and calcium signal on CT.

In a case series of children with ODD, Teixeira et al. found 89% had concomitant PHOMS in the same eye located predominantly in the nasal, superonasal and inferonasal sectors similar to the distribution of ODD.^37^ However, only ODD were found to harbour co‐localised sector abnormalities in RNFL thickness. Several case series of adults with ODD have shown a lower prevalence of accompanying PHOMS. In 23 adult eyes of isolated ODD (mean age = 43 years), Hamann et al. reported 10 (63%) had accompanying PHOMS.^9^ In another study of 76 eyes with isolated ODD (mean age = 38), Petzold et al. reported 18 (47%) with concurrent PHOMS.^7^ In a histological study of 31 eyes with ODD (mean age = 58 years, identified from 1713 enucleated globes), Skougaard et al. reported 5 (16%) with localised peripapillary axonal distensions resembling PHOMS.^38^ Further studies are needed to determine if the prevalence of PHOMS in ODD decreases with increasing age. This raises the question of whether PHOMS may spontaneously regress due to decompression of the ONH as ODD migrate to the surface and/or nerve fibres degenerate due to chronic axoplasmic stasis.

### 3.2 Papilloedema

**FIGURE 4:**
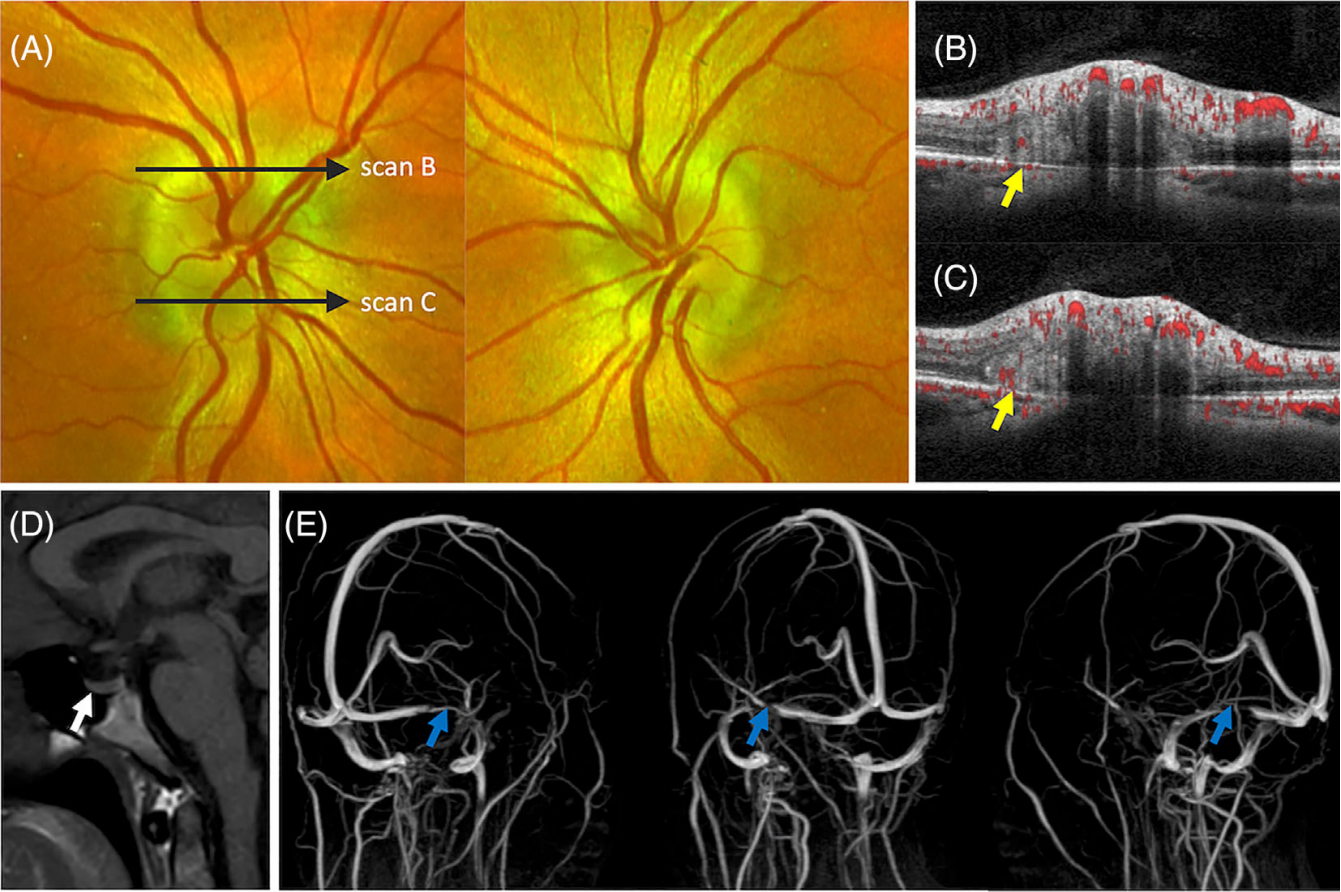
A 45‐year‐old obese female with idiopathic intracranial hypertension presented with headache, bilateral visual loss and papilloedema (A). The opening pressure was 27 cm water. OCT showed hyperreflective PHOMS with internal flow signals (yellow arrows) at the superior (B) and inferior (C) disc margins. Magnetic resonance imaging and venogram showed patulous optic sheath complexes, partially empty sella (white arrow) configuration (D) and bilateral flow gaps around the lateral angles of both transverse sinuses (blue arrows) indicating relative stenosis (E).

Bilateral optic disc swelling due to raised intracranial pressure (ICP) is known as papilloedema. Raised cerebrospinal fluid (CSF) pressure around the optic nerve alters the normal pressure gradient between the eye (intraocular pressure) and the retrolaminar tissue (ICP). The reversal of the trans‐lamina cribrosa pressure difference (TLCPD) results in orthograde axoplasmic flow stasis, disc swelling and vascular congestion. The most common cause of papilloedema is idiopathic intracranial hypertension (IIH), defined by the combination of raised intracranial pressure, without hydrocephalus or mass lesion, with a normal composition of the CSF and an absence of an underlying cause found.^39^ The age‐adjusted and gender‐adjusted incidence was reported to be 2.4 per 100 000 person‐years (during 2002–2014).^40^ The increasing incidence has been attributed to a higher prevalence of obesity.^41^ A previous histological study of papilloedema revealed distended and vacuolated axons in the prelaminar and peripapillary regions. Nerves fibres were also seen to bulge laterally over the rim of the Bruch's membrane opening as a fold that pushes the peripapillary retinal layers centrifugally and inwards without distorting the Bruch's membrane topography.^10^, ^42^ This peripapillary configuration seen histologically resembles the circumferential PHOMS observed around the disc margin in a case of IIH‐related papilloedema described by Alexander Fraser & Hamann.^10^, ^43^ Figure 4 illustrates a case of papilloedema associated with PHOMS demonstrating intrinsic vascular signals on OCTA. A small cystic lesion is occasionally seen between PHOMS and the displaced inner retinal layers (Figure 4C).

Wibroe described the prevalence of PHOMS in a cohort of patients with IIH‐related papilloedema.^44^ Among 18 patients with EDI‐OCT available at the time of diagnosis (acute presentation), 16 (89%) had PHOMS, all of which persisted at follow up. In 32 IIH patients (median age = 30 years) at follow up (range: 2 months to 7 years), 26 (81%) had PHOMS. In a sub analysis, the presence or absence of PHOMS did not influence RNFL thickness or ganglion cell layer volume. Another study of 13 subjects (mean age = 30 years) with IIH, 8 (62%) were found to have PHOMS.^7^ It was not clear if these patients had imaging performed at their initial presentation or after resolution of disc swelling following successful treatment. Alexander Fraser et al. showed a reduction in the size of PHOMS in a patient with IIH at 6 months after weight loss and acetazolamide treatment.^2^ However, there has been no prospective treatment study reporting the prevalence and natural history of PHOMS following treatment of IIH. Furthermore, it has not been possible to determine if PHOMS pre‐dates the onset of papilloedema as no historical disc imaging was performed prior to the onset of IIH. It has been hypothesized that PHOMS may also be observed in astronauts returning from long‐duration missions to the International Space Station due to impaired glymphatic efflux of ocular fluid into the optic nerve.^45^ However, PHOMS were not reported in a previous series of spaceflight associated neuro‐ocular syndromes.^46^, ^47^, ^48^

### 3.3 Anterior ischaemic optic neuropathy

**FIGURE 5:**
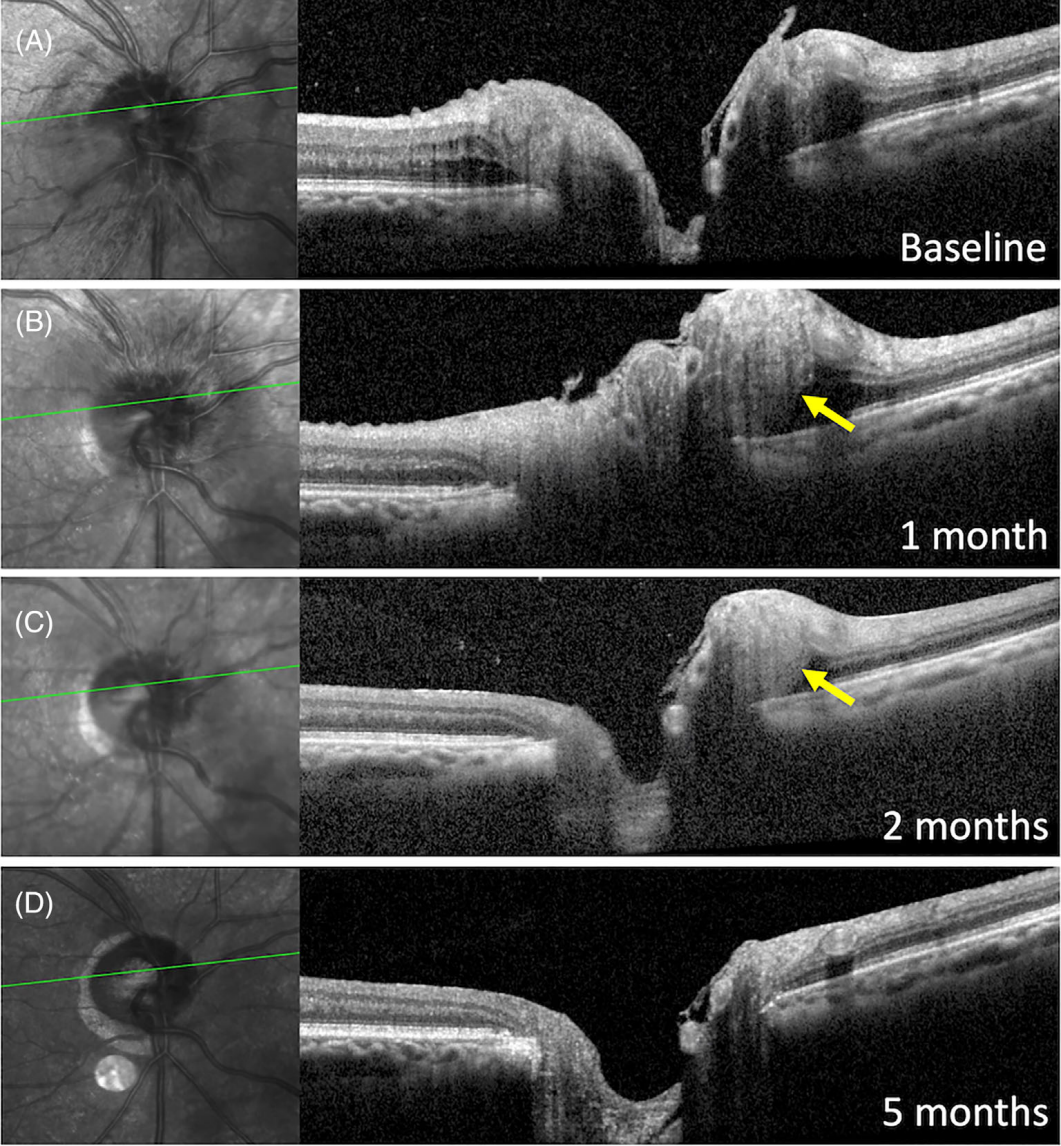
A right non‐arteritic anterior ischaemic optic neuropathy developed in a 68‐year‐old male at 2 months post uneventful routine cataract surgery. Disc elevation with intraretinal fluid was noted in the early phase (A). By 1 month, his vision had declined from 6/9 to 6/30 with the development of nerve fibre herniation nasally (B). At 2 months, there was a reduction in disc swelling with persistence of PHOMS and recovery of vision to 6/19. By 5 months, the oedema and PHOMS had completely resolved, and visual acuity remained stable at 6/19 (D). He had no systemic symptoms of giant cell arteritis and all inflammatory markers and full blood count were within normal limits.

In those over 50 years, AION is the most common acute optic nerve disease with 90% being nonarteritic (NA) in nature.^49^ The age‐adjusted annual incidence has been reported to be as high as 10.2 per 100 000 (during 1981–1990).^50^ Whilst occlusion of the small vessels supplying the retrolaminar region of the ONH has been accepted as the final event leading to AION, the relative contribution of multiple systemic and local anatomical risk factors continues to be debated.^51^ NA‐AION most commonly arises in an eye with a small crowded disc with or without ODD. Systemic risk factors include hypertension (seen in 50%) and diabetes mellitus (present in 25%). Other proposed systemic factors are obstructive sleep apnoea, cigarette smoking, nocturnal hypotension, use of amiodarone, phosphodiesterase type 5 inhibitors and vasopressors or vasoconstrictor medications.^49^ In younger NA‐AION patients, systemic risk factors are often absent and optic disc crowding due to ODD is frequently observed.^9^ Histology of a patient with a ruptured cardia papillary muscle and subsequent profound hypotension which resulted in visual loss 10 days prior to death showed optic nerve swelling with peripapillary crowding of the retina resembling PHOMS.^13^ Figure 5 illustrates the development and resolution of PHOMS in association with NA‐AION over a 5 month period as the disc oedema spontaneously resolved.

Hamann et al. reported ODD present in 51% of NA‐AION eyes in patients 50 years or younger. Of the unaffected fellow eyes 43% had ODD. Just over half of these young NA‐AION patients had ODD in one (29%) or both (71%) eyes. Among the 74 eyes with NA‐AION, 23 (31%) had PHOMS. The prevalence was higher if ODD was also present (54% with vs. 28% without ODD). In another case series, 5 of 9 (56%) patients with NA‐AION were found to have PHOMS but most of these (6 of 9) had co‐existing ODD.^52^ There are no data on the frequency of PHOMS in older patients with NA‐AION or arteritic‐AION. Whether PHOMS occurs in the setting of AION and disappears with the resolution of disc oedema remains to be determined.

### 3.4 Tilted disc syndrome

**FIGURE 6:**
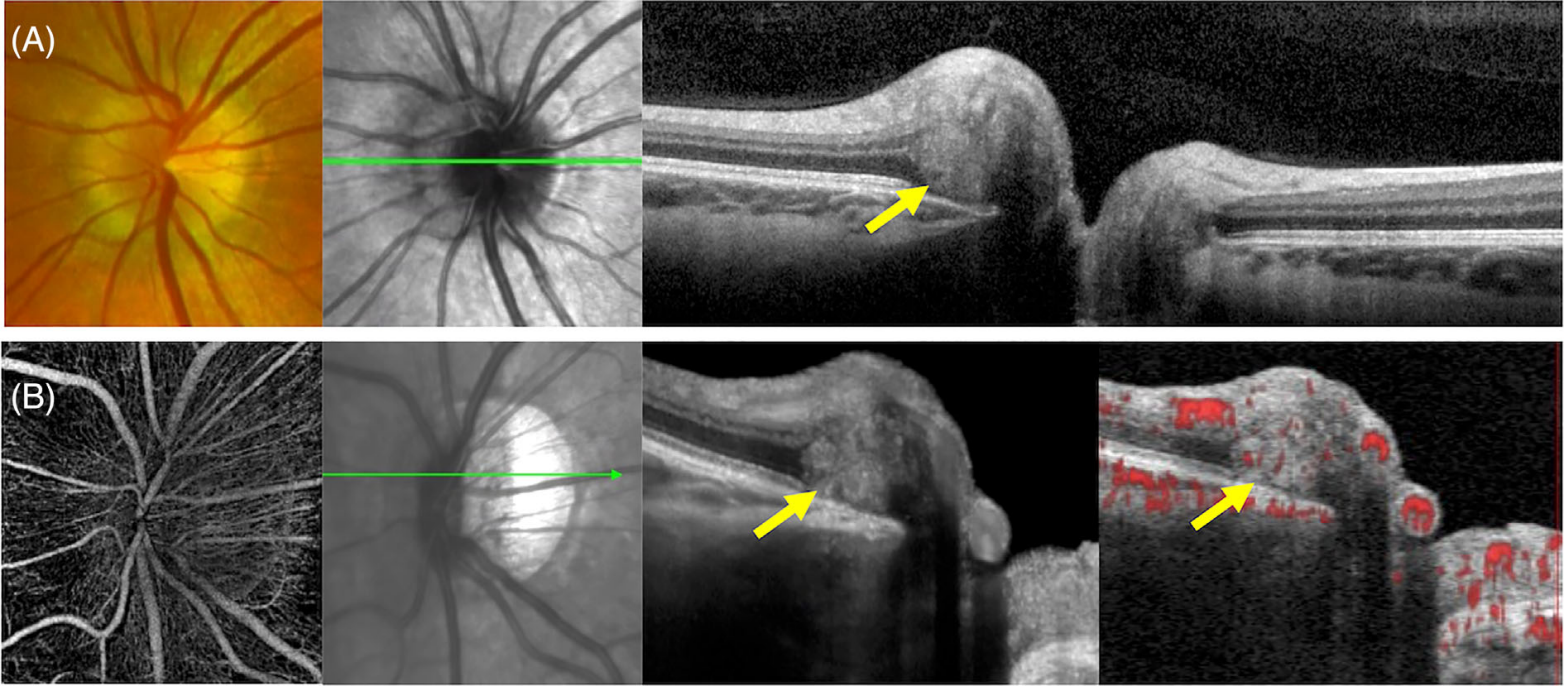
A tilted disc in a 17‐year‐old female with a moderate myopic refraction (−3.00 D) (A) and a 61‐year‐old female with a high myopic refraction (−7.50 D) (B). Both are associated with nasal peripapillary hyperreflective ovoid mass‐like structures (PHOMS). Acute bending of the optic nerve fibres is seen on the nasal disc margin leading to localised axoplasmic stasis and herniation (yellow arrow). OCTA showed internal vascular signals.

Torsion and tilting of the ONH were previously considered congenital anomalies but are now also recognised in progressive myopia. In an ovalised ONH, a torted disc is defined by >15° of rotation of longest axis from the vertical meridian^53^ whilst a tilted disc is defined by a ratio of the minimum to maximum optic disc diameter <0.75.^54^ The prevalence of torted and tilted discs were reported to be 64.7% and 3.5%, respectively, whilst 2.4% had both torted and tilted discs in a population study of 739 Chinese subjects in Singapore.^55^ The majority (88.5%) of tilted discs had a myopic refractive error. In the Blue Mountains Eye Study, a torted disc was found in 1.6% of the 3654 participants with myopia and in 66.2% of the eyes with tilted discs. Associated features in tilted discs were astigmatism (93.5%), situs inversus of retinal vessels (70.1%), beta‐peripapillary atrophy (64.9%), strabismus (30.4%), visual field defects (19.4%) and posterior staphyloma (18.2%).^56^ Whilst incomplete closure of the optic fissure is thought to be the mechanism of congenital disc tilting (short axis pointing inferiorly or nasally) and torsion,^57^ a nasal shifting of the temporal disc margin has been proposed as the mechanism behind myopic disc tilting (short axis pointing temporally).^58^ Histopathology of tilted discs has shown lateral bulging of the optic nerve fibres attributed to axoplasmic stasis secondary to (a) impingement from Bruch's membrane protruding into the optic nerve and (b) nasal dragging of the lamina cribrosa relative to the Bruch's membrane opening.^2^ Figure 6 demonstrates a vascularised PHOMS associated with a myopic temporal disc tilt. In this case PHOMS arises at the nasal margin where the optic nerve makes a sharp bend around the protruding Bruch's membrane.

Pichi et al. first described herniation of the nerve fibres above the protrusion of Bruch's membrane and the choroid from the nasal side of the disc in 15 of 38 (39%) eyes or 14 of 20 (70%) patients with tilted discs.^5^ The OCT features described by Pichi et al. were akin to the later named PHOMS by Malmqvist et al. In a 1‐year follow up examination of the 15 eyes with PHOMS‐like lesions, 7 eyes (47%) were reported to decrease in thickness. It was not known whether this reduction was significant enough to cause a permanent visual field defect. Nevertheless, they did find 37% of eyes with a baseline field defect that was initially reversible became only partially correctable with maximum myopic refraction at 1 year follow‐up. Petzold et al. reported 36 of 81 (44%) subjects with an anomalous disc to exhibit PHOMS on OCT.^7^, ^19^ They did not describe what type of disc anomalies were included in this cohort. Others have reported the presence of PHOMS in a myopic tilted disc complicated by a dome‐shaped macula^59^ and the emergence of PHOMS as the optic disc became ovalised with myopic progression.^60^ It remains to be determined if there are differences in PHOMS associated with congenital disc torsion and tilting versus acquired myopic disc ‘tilting’ and whether PHOMS is a risk factor for the future development of visual field defects associated with disc tilting.

### 3.5 Inflammatory demyelinating disorders

**FIGURE 7:**
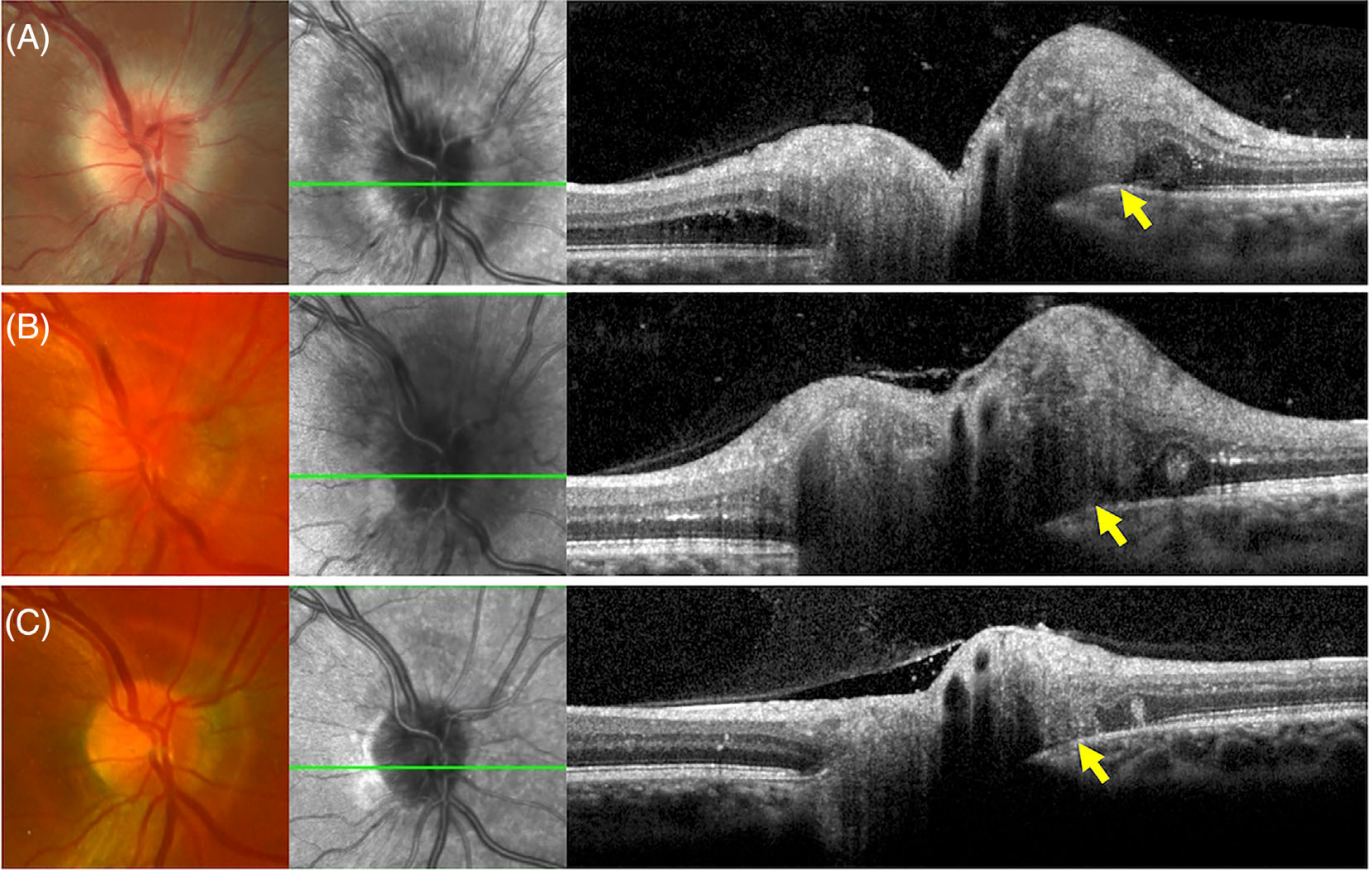
A 28‐year‐old male presented with a right subtle blurry patch of vision accompanied by pain on eye movement. Disc oedema with peripapillary intraretinal fluid and nerve fibre swelling was noted (A). A peripapillary hyperreflective ovoid mass‐like structure became more prominent 2 weeks later (B). By 3 months, vision had recovered with resolution of the disc oedema but persistence of the PHOMS (C). Throughout the course of this episode, visual acuity remained 6/6 and magnetic resonance imaging pre and post gadolinium showed no abnormal signal or enhancing mass along the right optic nerve sheath complex. There was no evidence of intracranial demyelination.

Multiple sclerosis (MS) is a CNS inflammatory demyelinating disorder characterised by sensory and motor dysfunction and loss resulting from immune‐mediated demyelination and subsequent axonal damage. The prevalence of MS in Hobart (latitude of 42.5° S) was 147 per 100 000 in 2019 and the age‐standardised incidence was 6.1 per 100 000 person‐years (during 2009–2019).^61^ The increasing prevalence has been attributed to decreased mortality, increased longevity and increased incidence.^62^ In addition to the classical MS, there are various subtypes of CNS demyelinating syndromes which have a particular predilection for optic nerve involvement such as neuromyelitis optica (NMO) and recurrent isolated optic neuritis. As the optic nerve is an extension of the CNS, previous studies have hypothesized reduced RNFL thickness may be a biomarker for axonal loss in the CNS even in the absence of clinical optic neuritis.^63^, ^64^ However, more recent cross‐sectional and longitudinal studies showed no statistically significant difference in RNFL thinning in MS patients without a prior history of optic neuritis as compared to controls.^65^, ^66^ Although histopathology of human optic neuritis is lacking, there is evidence from a guinea pig model of autoimmune optic neuritis demonstrating swollen axons displaced into the peripapillary retinal tissue forming an S‐shaped bulge resembling PHOMS.^16^ An example of persistent PHOMS in association with resolving isolated optic neuritis is shown in Figure 7.

Petzold et al. explored the association of PHOMS in MS cases with and without a history of optic neuritis.^7^ Overall, 16% of MS patients exhibited PHOMS on OCT. These lesions may have developed de novo or were observed at baseline with stability or a slight increase in their size during follow up. A history of optic neuritis did not seem to influence the prevalence of PHOMS. In the same study, a control cohort of 16 isolated optic neuritis cases without MS had a 19% prevalence of PHOMS. Interestingly, lower fractional anisotropy (a measure between 0 and 1 that describes directionality of diffusion) in the optic radiations was associated with absence of PHOMS. Although lower anisotropy typically represents nerve bundle atrophy, this measure does not have pathological specificity. In another study, PHOMS was found in 18% of 649 patients with early relapsing–remitting MS. There was no association between presence of PHOMS and age, sex and the Expanded Disability Status Scale.^8^ Eyes with a history of optic neuritis has a higher frequency of PHOMS than eyes without (16.8% vs. 12.7%). In a younger cohort of patients with MS of a duration of 2–3 years, the prevalence of PHOMS was 18.5% and 19.7% for relapsing–remitting and primary progressive MS, respectively. Longer disease duration in the primary progressive MS group was associated with PHOMS. There have been no reports of PHOMS in other variants of MS or any analysis of the change in PHOMS size following treatment. PHOMS in MS has been thought to be related to axoplasmic stasis, glymphatic system impairment and reversal of the translaminar pressure gradient. Future work is required to investigate if PHOMS is a useful imaging biomarker for MS subtypes, disease course or response to therapy.

### 3.6 Other retinal disorders and orbital disease associations

**FIGURE 8:**
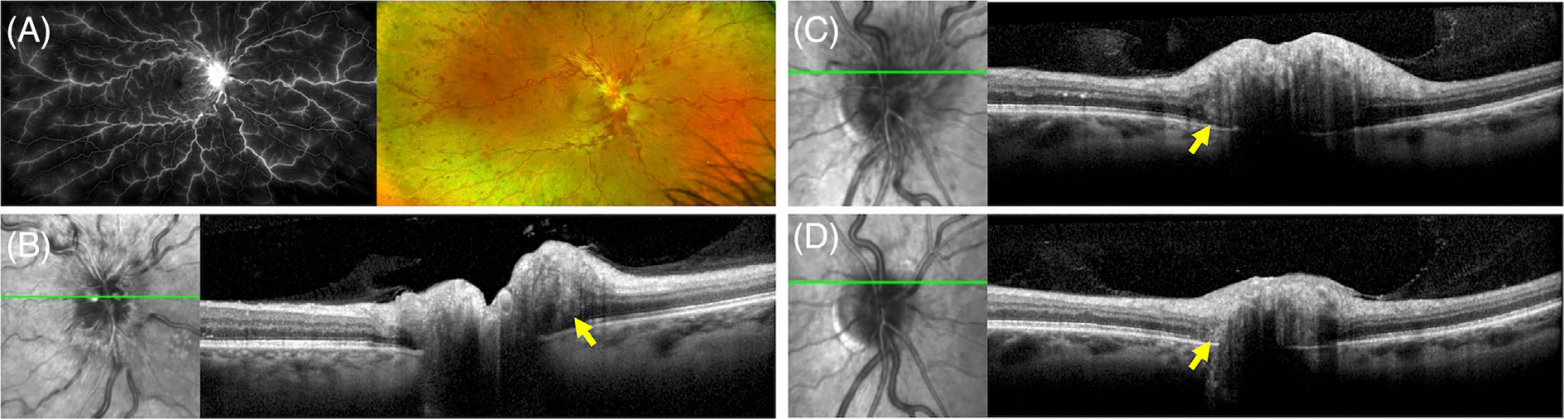
A right nonischaemic central retinal vein occlusion in a 53‐year‐old female (A) with accompanying disc oedema. OCT through the disc showed peripapillary hyperreflective ovoid mass‐like structures (PHOMS) nasally as marked by yellow arrows (B). A 42‐year‐old male with type 2 diabetes mellitus presented with right disc oedema with no visual loss and normal magnetic resonance imaging (MRI) (C). The presumed diabetic papillopathy resolved spontaneously 12 months later with partial regression of PHOMS as marked by the yellow arrows (D).

**FIGURE 9:**
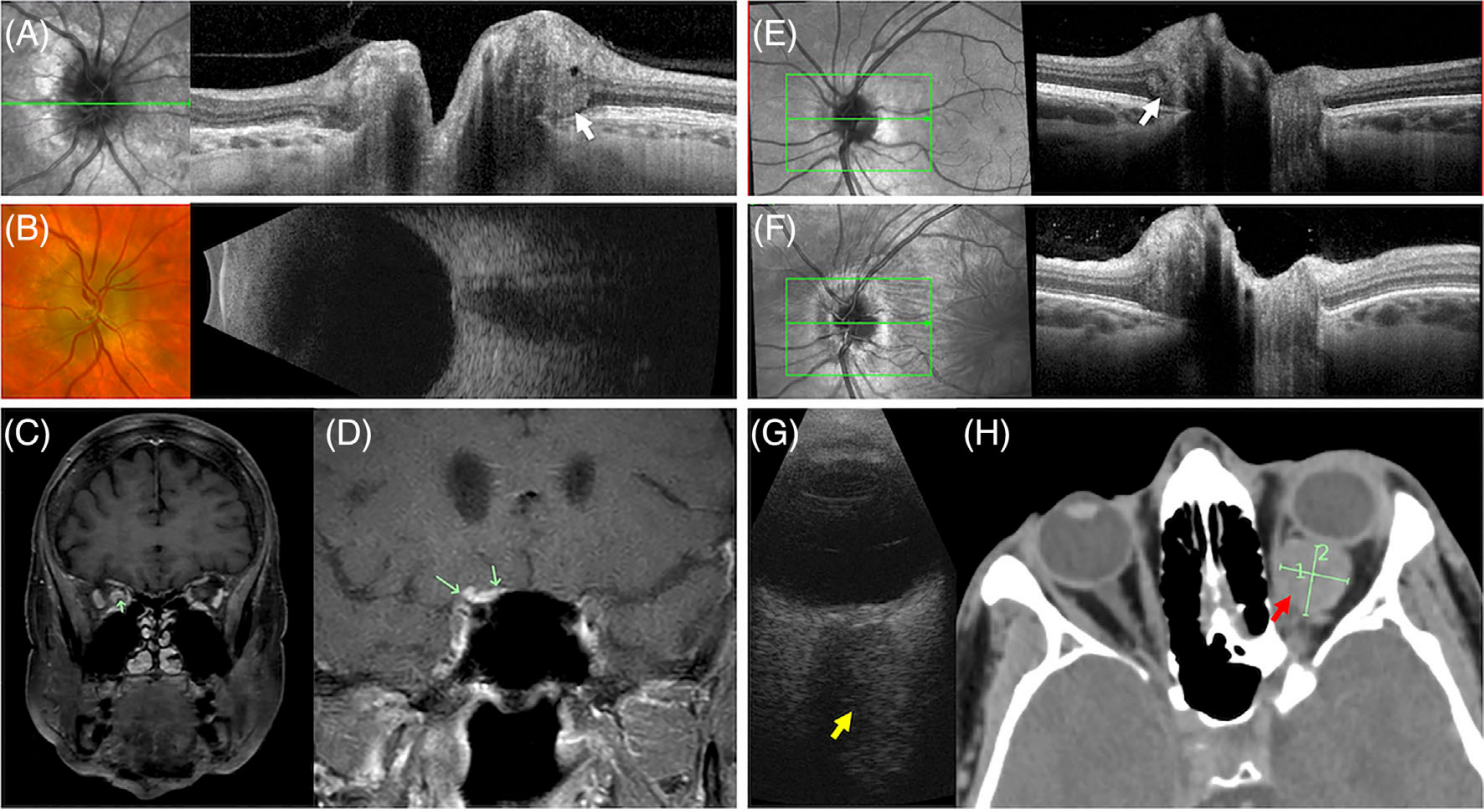
A 66‐year‐old female was diagnosed with optic nerve sheath meningioma 20 years prior when she presented with optic nerve elevation (A‐D). OCT scan through the chronically elevated disc showed PHOMS (white arrow) with intraretinal cystic change at the inner retinal tissue border (A). An elevated optic nerve head was seen on ultrasonography (B). MRI showed circumferential optic nerve sheath thickening and enhancement (green arrow in right orbit, C) with extension of the meningioma posteriorly along the planum sphenoidale attached to a small nodular component (green arrows) along the superior margin of the anterior clinoid process (D). A 30‐year‐old male had a routine optic disc OCT imaging 2.5 years prior to presentation with painful gaze and gaze‐evoked amaurosis (E‐H). The PHOMS that was seen 2.5 years prior (E) but not 11 years prior had enlarged with development of symptoms and new choroidal folds (F). B‐scan ultrasound showed flattening of the posterior globe by an echogenic mass lesion (yellow arrow) adjacent to the optic nerve (G). On computed tomography, the 17 × 17 × 15 mm (markers) intraconal mass (red arrow) that was isodense to the muscle was abutting and flattening the posterior globe and displacing the optic nerve sheath complex inferolaterally (H). Further investigation with magnetic resonance imaging confirmed a low flow vascular malformation.

Given PHOMS is a sign of axoplasmic stasis, it is not surprising that other retinal disorders and orbital diseases which cause compartment syndrome at the ONH or optic nerve compression may predispose to its formation. Dai et al. reported 3 of 25 patients with CRVO and 3 of 14 patients with branch‐RVO (BRVO) had accompanying PHOMS.^52^ It remains unclear how a BRVO can lead to ONH compartment syndrome unless this was a hemi‐central RVO or the PHOMS was incidental and unrelated to the BRVO. Prior to PHOMS being defined by the ODDS consortium, Lee et al published observations of a ‘buried ODD’ in cases of optic disc melanocytoma, optic nerve meningioma and optic nerve glioma.^6^ These lesions all share a common endpoint of axoplamic stasis. It remains to be investigated if other causes of disc swelling such as diabetic papillopathy, malignant hypertension, posterior uveitis, posterior scleritis, thyroid orbitopathy, drug toxicities and hereditary optic neuropathies (Leber hereditary optic neuropathy and Retinal dystrophy, optic nerve edema, splenomegaly, anhidrosis, and migraine headache [ROSAH] syndrome) may also manifest PHOMS. Figure 8 illustrates PHOMS in association with a CRVO and diabetic papillopathy. Figure 9 illustrates two cases of optic nerve compression with PHOMS. A longstanding optic nerve sheath meningioma of 20 years duration without visual loss can manifest as chronic disc margin elevation due to PHOMS (Figure 9A–D). In a patient with asymptomatic PHOMS, further investigation may be required to exclude optic nerve compression by an orbital lesion. As illustrated in Figure 9E–H, a patient with asymptomatic PHOMS found incidentally returned 2.5 years later with painful gaze‐evoked vision loss due to an enlarging cavernous venous malformation. Although orbital imaging wasn't performed 2.5 years prior, the absence of PHOMS at the disc margin 11 years prior suggests the orbital tumour was present and already compromising axoplasmic flow without causing visual symptoms.

## 4 CONCLUDING REMARKS

Redefining peripapillary hyperreflective ‘buried ODD’ as PHOMS has enhanced our understanding of a common pathway in ONH compartment syndrome and axoplasmic stasis due to a wide range of CNS, optic nerve and retinal diseases. Multimodal imaging including EDI‐OCT, NIR, FAF and US are essential for both the characterisation of PHOMS and determining the underlying disease process precipitating the herniation of nerve fibres. Incorporating a high density optic disc raster and radial scans as set out by the ODDS consortium into our routine ONH assessment will enhance the detection rate and allow for a longitudinal analysis of PHOMS evolution and regression. The inclusion of visual field or microperimetric analysis in diseases with a high frequency of PHOMS may help to determine the relevance of nerve fibre herniation as a potential risk factor for permanent axonal loss. PHOMS as biomarker of optic nerve compartment syndrome should be considered as an exploratory endpoint measure in future clinical trials examining the treatment of papilloedema, AION, myopia progression, CNS demyelination and other diseases that lead to optic disc oedema. Future histological studies of eyes that have clinical evidence of PHOMS is essential for confirming the hypothesis that this toroidal hyperreflective structure originates from herniated optic nerve fibres as a response to ONH compartment syndrome.

## FUNDING INFORMATION

This study was supported by the National Health & Medical Research Council of Australia (project and fellowship grant number: GNT1116360 (FKC), GNT1188694 (FKC), GNT1054712 (FKC) and MRF1142962 (FKC)), the McCusker Foundation (FKC), the Miocevich Retina Fellowship (RCHJ) and Channel 7 Telethon Trust (FKC).

## CONFLICT OF INTEREST

The authors declare no conflict of interest.

## ETHICS STATEMENT

Patients have given consent for these images to be used in a prospective study. Ethics approval was obtained from the Human Ethics Office of Research Enterprise, the University of Western Australia (RA/4/1/7916, RA/4/20/5454, RA/4/1/8932 and 2021/ET000151) and Sir Charles Gairdner Hospital Human Research Ethics Committee (2001‐053).
